# Commentary: The flexibility of SABRE, a new quantitative receptor function model, in fitting challenging concentration-effect data

**DOI:** 10.3389/fphar.2025.1675039

**Published:** 2025-09-23

**Authors:** Peter Buchwald

**Affiliations:** ^1^ Diabetes Research Institute, Miller School of Medicine, University of Miami, Miami, FL, United States; ^2^ Department of Molecular and Cellular Pharmacology, Miller School of Medicine, University of Miami, Miami, FL, United States

**Keywords:** SABRE model, operational model, Furchgott’s method, curve fitting, adenosine receptor, GraphPad Prism

## 1 Introduction

In their recent study, Olah et al. (2025) applied the SABRE (Signal Amplification, Binding affinity, and Receptor-activation Efficacy) model (Buchwald, 2019; Buchwald, 2020; Buchwald, 2022) to the analysis of their adenosine receptor response data, which had been previously measured (Gesztelyi et al., 2013) with three different agonists (NECA: 5′-(N-ethylcarboxamido) adenosine, CPA: N^6^-cyclopentyladenosine, and CHA: N^6^-cyclohexyladenosine) at seven concentrations ranging from 10^−10^ to 10^−4^ M, before (N) and after (X) partial-irreversible inactivation with FSCPX ((8-cyclopentyl-N^3^-[3-(4-(fluorosulfonyl)benzoyloxy)propyl]-N^1^-propylxanthine)). Unequivocal fitting of these data obtained at different receptor levels (i.e., Furchgott’s method) is particularly challenging because only a single inactivation level was used—one that resulted in no reduction of the maximal effect in any of the responses. Using an iterative approach involving four different fitting strategies, the authors concluded that “the SABRE model is at least as useful as two widely accepted older methods thought to have similar capabilities, the operational model of agonism and Furchgott’s method, even if the quality of the data to be evaluated is somewhat challenging” (Olah et al., 2025).

Although the authors used a detailed and careful approach, the final SABRE fit obtained is not the best unified fit that can be achieved. As highlighted in their article, “The first step in regression is to choose a proper model (equation),” and this necessarily involves choosing the right parameter setting. This is especially important with the SABRE model, since it was intentionally designed to be a general model with multiple parameters that can and should be restricted for specific cases as needed: “Its general form… can be reduced to consecutively nested, simplified forms for special cases of its parameters…, and these can be used on their own when adequate” (Buchwald, 2020). For the present case, this would mean three parameters that are the same across all data as they characterize the response (specifically, the Hill coefficient *n*, the pathway amplification *γ*, and the fraction of receptors inactivated *q*), and two parameters that are the same for each agonist as they characterize the agonists (specifically, the binding affinity constant *K*_d_ and the efficacy *ε*). This was achieved only in their final, fourth strategy; however, even there, it was not a single unified fit of the entire dataset, as it involved first fitting “the datasets generated with the same agonist” to obtain *K*_d_ estimates and then using these fixed “*K*_d_ values, provided by the third fitting strategy,” to perform “a six-model global fitting” (Olah et al., 2025). Thus, it is not a single unified fitting, as the *K*_d_ values are not estimated in the final step but are instead retained at the constant values obtained in the previous fit of the individual compound data.

SABRE has not yet been implemented in GraphPad Prism, the most widely used and powerful software program for nonlinear regression of pharmacological data, and the program used for these fittings; therefore, custom “user-defined equations” have to be used. Because GraphPad Prism, in its current form, only allows parameters that are individually fitted for each dataset (“no constraint”), restricted to a common value across all datasets (“shared value for all datasets”), fixed as a single constant value (“constant equal to”), or fixed as constant for each set (“dataset constant from column title”), its implementation for complex data involving multiple agonists and receptor levels is not straightforward. Thus, either separately defined equations must be used for each dataset (column), as was done in the study by Olah et al., (2025) (see Supplementary Material in Olah et al., (2025)), or a combination of custom ranges for one equation per compound *i* (with same *K*_d,*i*_ and the efficacy *ε*_*i*_) must be used, together with special column headers and corresponding conditional parameters for each inactivation *j* (to allow the same *q*_*j*_), as was done before to fit Furchgott-type data (e.g., Figure 4 in Buchwald, 2022).

## 2 Results

**FIGURE 1:**
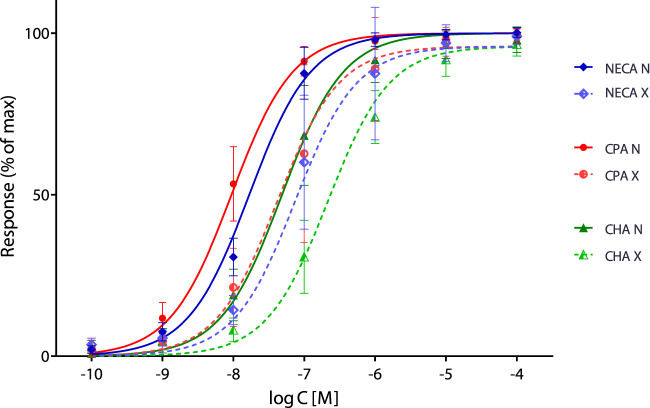
Full unified fit of the present data Olah et al. (2025) with SABRE, assuming a single pathway (with shared values for *n*, *γ*, and *q*) and three agonists, each with their own *K*_d_ (*K*_d,NECA_, *K*_d,CPA_, and *K*_d,CHA_) and *ε* (results shown for “approach 5” with *ε*_NECA_ = *ε*_CPA_ = *ε*_CHA_ = 1).

A single, unified fitting of this dataset can be achieved by using either of these SABRE implementations as a more correct “fifth” strategy—this has been performed in this study with the generous support of the authors, who provided their original data and models. Results obtained under the original assumption that all three agonists are full agonists (i.e., all efficacies are equal to 1: *ε*_*NECA*_ = *ε*_*CPA*_ = *ε*_*CHA*_ = 1) are presented in Figure 1 and Table 1. Although the fit improves only slightly (as indicated by the decrease in the global sum of squared errors, (SSE), from 20,459 to 18,354) and the parameter values do not change significantly, this constitutes a true unified fitting of all data, as all parameters are obtained within a single fit. Undeniably, fitting of these data remains challenging, and even with this unified SABRE fit (“approach 5”), the parameters cannot be fully separated: dependency values remain in the high or unacceptably high range (>0.9 and >0.99, respectively), with the sole exception of the fraction inactivated (*q*) value.

**TABLE 1:** Fit of the present data from Olah et al. (2025) with SABRE, assuming a single pathway (shared values for *n = 1*, *γ*, and *q*) and full agonists (*ε*_NECA_ = *ε*_CPA_ = *ε*_CHA_ = 1), each with their own *K*_d_ (*K*_d,NECA_, *K*_d,CPA_, and *K*_d,CHA_).

Parameters	NECA N	NECA X	CPA N	CPA X	CHA N	CHA X
SABRE published fit (“approach 4” from Olah et al. (2025))						
*n*	1					
*γ*	86.84 ± 6.34					
*q*		0.217 ± 0.022		0.217 ± 0.022		0.217 ± 0.022
*ε*	1					
log *K*_d_	−5.882 *(fixed)*		−5.927 *(fixed)*		−5.506 *(fixed)*	
global *r* ^2^	0.956					
global SSE	20,459					

Parameters	NECA N	NECA X	CPA N	CPA X	CHA N	CHA X
SABRE full unified fit (“approach 5”)						
*n*	1					
*γ*	85.43 ± 35.47					
*q*		0.217 ± 0.023		0.217 ± 0.023		0.217 ± 0.023
*ε*	1					
log *K*_d_	−5.843 ± 0.164		−6.094 ± 0.164		−5.381 ± 0.164	
global *r* ^2^	0.960					
global SSE	18,354					

With this implementation, even the three efficacies can be released and fitted, allowing for the possibility of partial agonism; however, this results in only a very minimal improvement in the overall fit (SSE of 18,142 vs. 18,354) and, due to the nature of the data, it leads to highly uncertain parameter values; therefore, it was not included here. Nevertheless, it is worth mentioning that this fit indicates that CPA and CHA may be less effective than NECA in producing this particular response. Although all three are typically assumed to be full agonists, there are assays indicating possible functional selectivity and cases in which “NECA was the most efficacious agonist … compared to the other agonists, although it had the lowest potency” (Verzijl and Ijzerman, 2011). The difference in efficacies could also explain why the *q* value obtained for NECA via the classic Furchgott’s method differs from those of CPA and CHA (0.22 vs. 0.11–0.13) or why the corresponding pharmacological shift ratios (*K*_d_/EC_50_) are also 5–10 fold different (Gesztelyi et al., 2013).

## 3 Discussion

The main challenge with this dataset is that it does not allow for adequate separation of efficacies, binding affinities, and amplification due to the use of only a single inactivation level—one that resulted in no reduction of the maximal effect in any of the responses. As noted by Olah et al. (2025), “For a reliable evaluation, the maximal effect after partial-irreversible receptor inactivation is thought to have to be significantly smaller than the original maximal effect … .” Nevertheless, SABRE is still unique in its ability to allow estimation of the inactivation level (*q*), receptor reserve/signal amplification (*γ*), and compound potencies (*K*_d_) and efficacies (*ε*) in a single fit of the entire dataset—something that cannot be achieved with the operational model of agonism (Black and Leff) or the classical Furchgott’s method.
